# Supplementary material for: CD5L as a promising biological therapeutic for treating sepsis
Source: Nat Commun. 2024 May 15;15:4119. doi: 10.1038/s41467-024-48360-8 (PMC11096381; doi:10.1038/s41467-024-48360-8)
Supplement: Supplementary file 1 — Supplementary Information [file 41467_2024_48360_MOESM1_ESM.pdf]

Figure S1

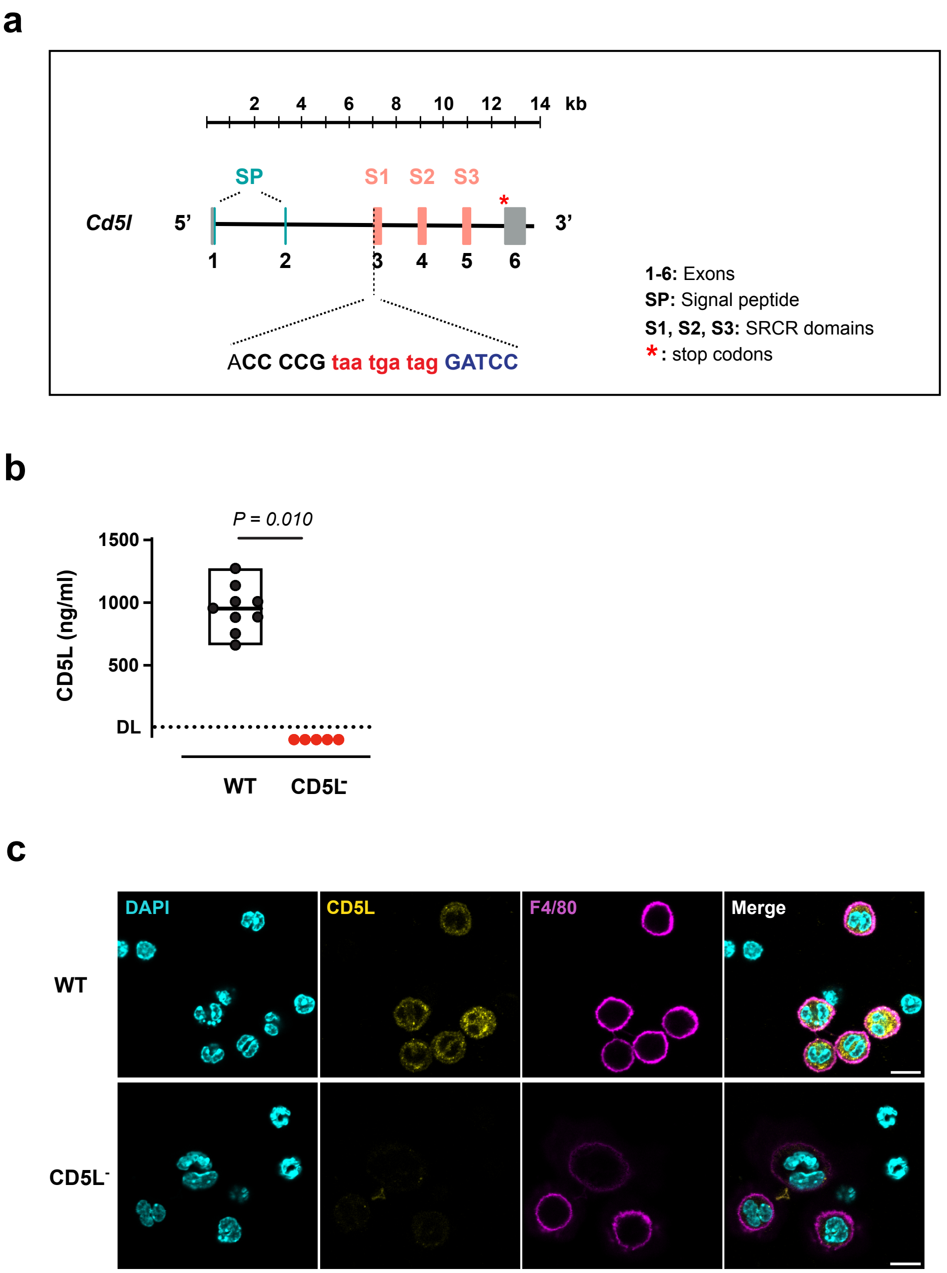

Fig. S1. Generation of CD5L⁻ mice and analysis of expression. a CD5L⁻ mice were generated by CRISPR-Cas9 engineering. Schematic representation of the genome editing strategy to silence *Cd5l* expression by inserting three in-frame stop codons (red) and a frame shift (blue). **b** Quantification of CD5L by ELISA in sera of WT and CD5L⁻ mice. DL: detection limit (6.25 pg/ml). Data from at least 3 independent experiments analyzed by two-tailed Wilcoxon signed-rank test. *n*= 9 (WT) and *n*=5 (CD5L⁻) animals/group. Floating bars show the minimum, average (line), and maximum values within each group. **c** Peritoneal macrophages from WT or CD5L⁻ healthy mice are identified in magenta through staining with anti-mouse F4/80 mAb and intracellular CD5L (yellow) was detected in Triton X-100-permeabilized cells. DAPI was used as nuclear counterstaining (cyan). Scale bar: 10 μm.

Figure S2

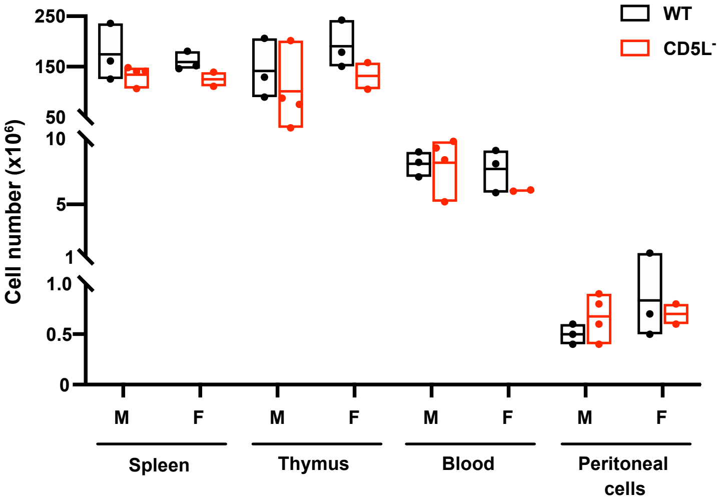

Fig. S2. Immunophenotyping of CD5L⁻ mice. Total leukocyte numbers in the spleen, thymus, peripheral blood and peritoneal cavity of C57BL/6 WT and CD5L⁻ naïve male (M) and female (F) mice. n= 3 animals/group except in CD5L⁻ females (n=2 animals). Floating bars show the minimum, average (line), and maximum values within each group.

Figure S3

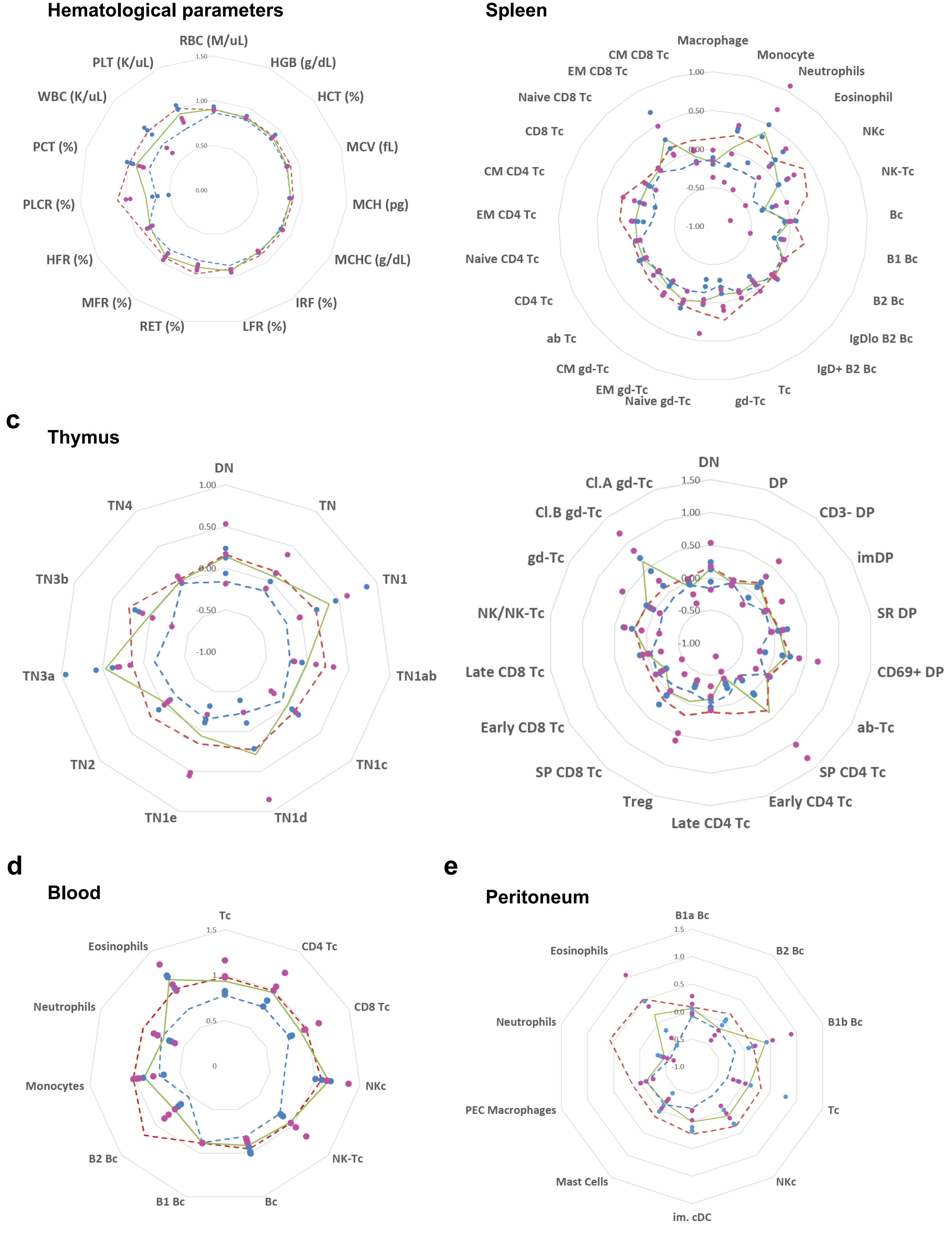
a
Hematological parameters
RBC (M/uL)
HGB (g/dL)
HCT (%)
MCV (fL)
MCH (pg)
MCHC (g/dL)
IRF (%)
LFR (%)
RET (%)
MFR (%)
HFR (%)
PLCR (%)
PCT (%)
WBC (K/uL)
PLT (K/uL)
1.50
1.00
0.50
0.00
b
Spleen
Macrophage
Monocyte
Neutrophils
Eosinophil
NKc
NK-Tc
Bc
B1 Bc
B2 Bc
IgDlo B2 Bc
IgD+ B2 Bc
Tc
gd-Tc
Naive gd-Tc
EM gd-Tc
CM gd-Tc
ab Tc
CD4 Tc
Naive CD4 Tc
EM CD4 Tc
CM CD4 Tc
CD8 Tc
Naive CD8 Tc
EM CD8 Tc
CM CD8 Tc
1.00
0.50
0.00
-0.50
-1.00
c
Thymus
DN
TN
TN1
TN1ab
TN1c
TN1d
TN1e
TN2
TN3a
TN3b
TN4
1.00
0.50
0.00
-0.50
-1.00
DN
DP
CD3- DP
imDP
SR DP
CD69+ DP
ab-Tc
SP CD4 Tc
Early CD4 Tc
Late CD4 Tc
Treg
SP CD8 Tc
Early CD8 Tc
Late CD8 Tc
NK/NK-Tc
gd-Tc
Cl.B gd-Tc
Cl.A gd-Tc
1.50
1.00
0.50
0.00
-0.50
-1.00
d
Blood
Tc
CD4 Tc
CD8 Tc
NKc
NK-Tc
Bc
B1 Bc
B2 Bc
Monocytes
Neutrophils
Eosinophils
1.5
1
0.5
0
e
Peritoneum
B1a Bc
B2 Bc
B1b Bc
Tc
NKc
im. cDC
Mast Cells
PEC Macrophages
Neutrophils
Eosinophils
1.5
1.0
0.5
0.0
-0.5
-1.0

Fig. S3. Immunophenotyping of CD5L$^-$ mice. FC Radar plots of hematological parameters (**a**), and leukocyte proportion variations between CD5L$^-$ and control animals in spleen (**b**), thymus (**c**), blood (**d**), and peritoneal cavity (**e**). EM: effector memory; CM: central memory; TN: triple negative; SP: single positive; DP: double positive; im: immature; ab: $\alpha\beta$ T cells; gd: $\gamma\delta$ T cells; Bc: B cells, Tc: T cells; NKc: NK cells; PEC: peritoneal resident macrophages. Each circle (blue: male; pink: female) represents a value obtained in independent CD5L$^-$ mice (value measured in (CD5L$^-$ −n)/(Mean WT)-1). Green line: average variation of subset proportion in CD5L$^-$ compared with WT animals [(Mean CD5L$^-$)/(Mean WT)-1]; blue dashed line: lower limit of WT value dispersion [(Mean WT - 1 SD)/(Mean WT)-1]; red dashed line: higher limit of WT value dispersion [(Mean WT + 1 SD)/(Mean WT)-1]. Populations were defined according to the expression of markers detailed in Supplementary Data 2. Values expressed in asinh ratio. This high content analysis exemplifies the absence of major leukocyte changes in CD5L$^-$ mice.

Figure S4

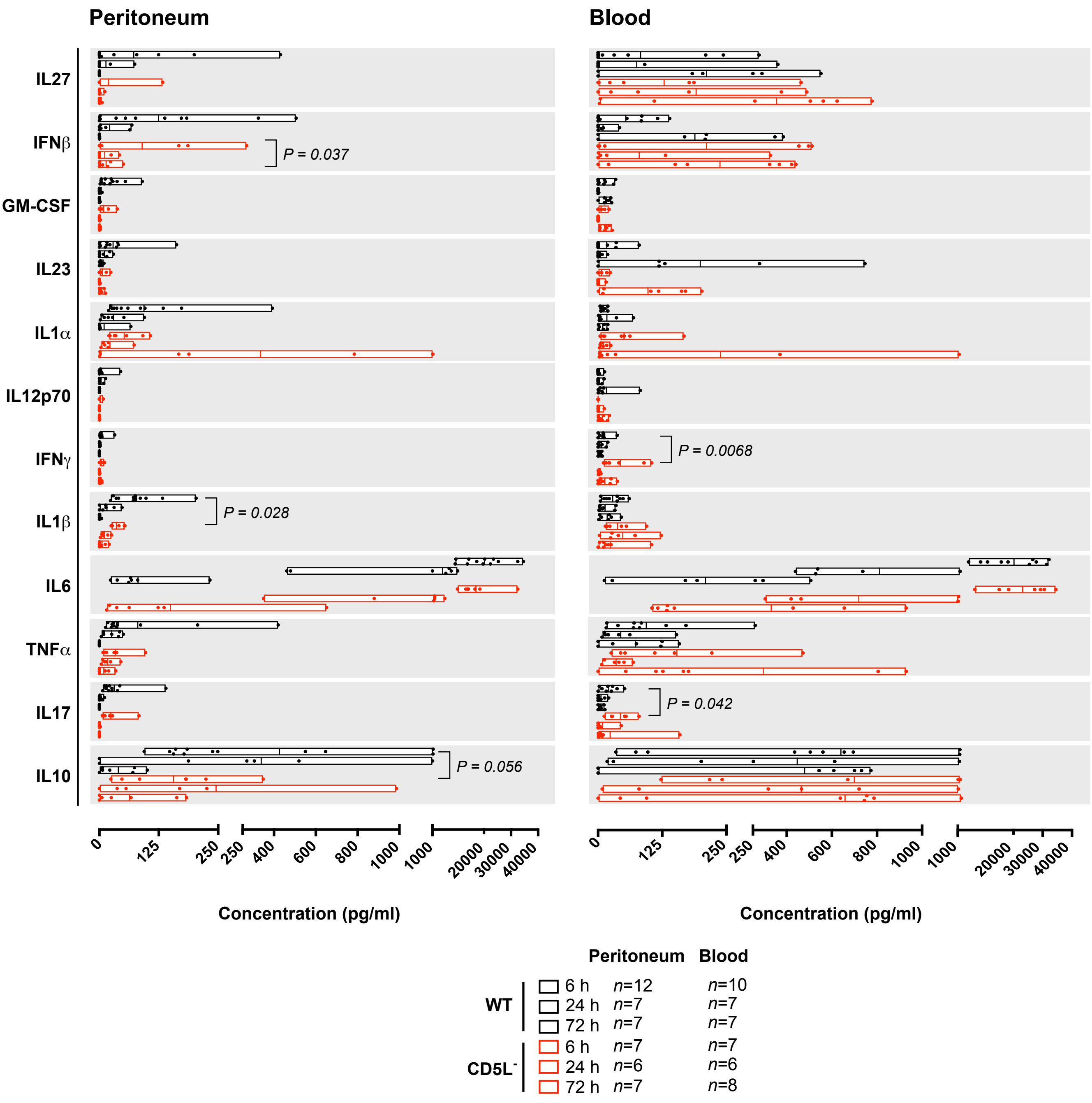

Fig. S4. Local and systemic cytokine profiles in WT and CD5L⁻ mice after mid-grade CLP. The indicated cytokines were quantified by bead-based multiplex immunoassay on samples from the peritoneal cavity (left) and blood serum (right) of WT and CD5L⁻ mice, 6, 24 and 72 h after mid-grade CLP. Pooled data are from at least 2 independent experiments, and statistical comparisons were drawn after performing two-tailed Mann-Whitney test. Floating bars show the minimum, average (line), and maximum values. The number of animals (*n*) in each group is indicated.

Figure S5

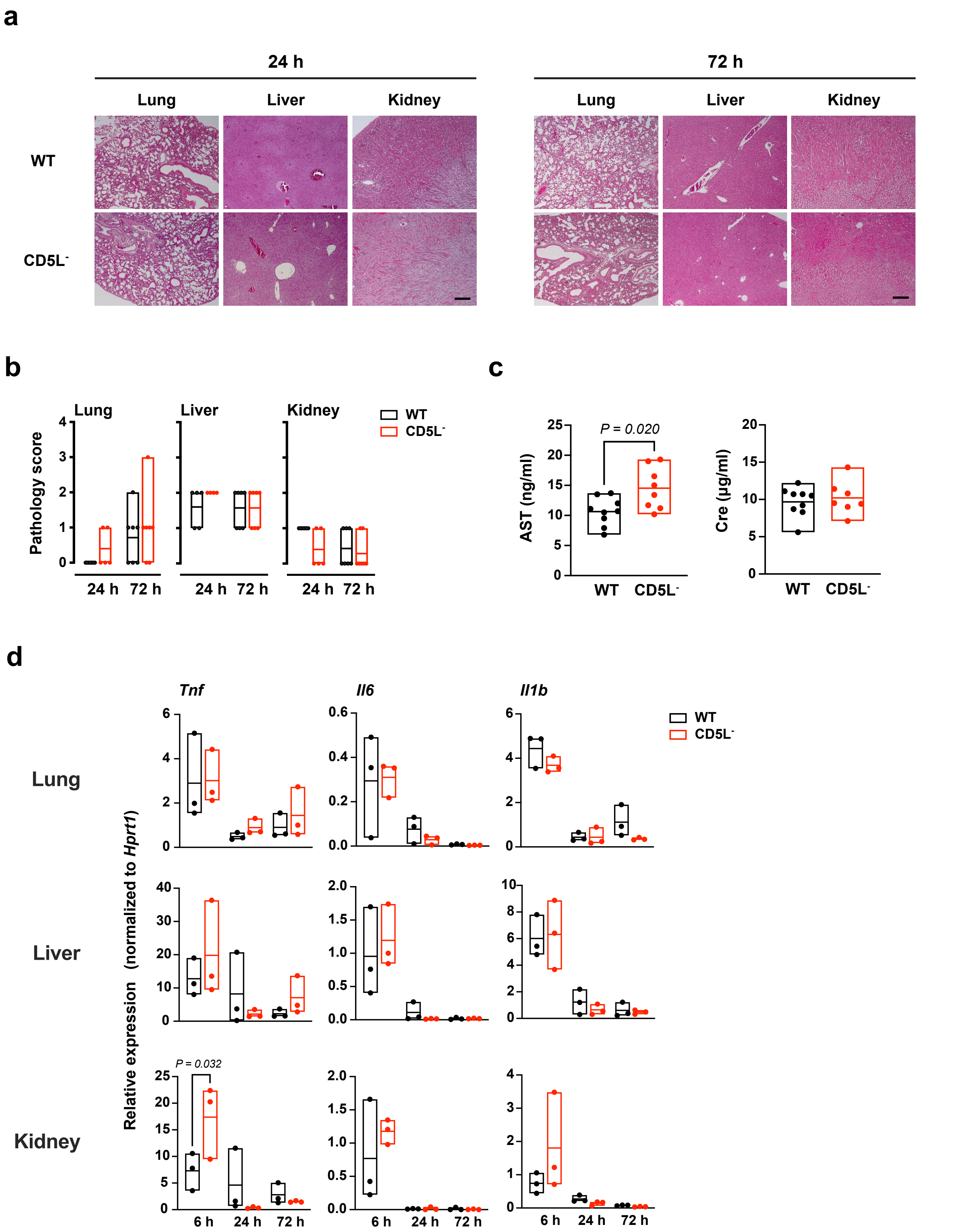
a
24 h
72 h
Lung
Liver
Kidney
WT
CD5L-
b
Pathology score
Lung
Liver
Kidney
WT
CD5L-
24 h
72 h
c
AST (ng/ml)
P = 0.020
Cre (μg/ml)
WT
CD5L-
d
Tnf
Il6
Il1b
Lung
Liver
Kidney
Relative expression (normalized to Hprt1)
P = 0.032
6 h
24 h
72 h

Fig. S5. Pathophysiology analysis of systemic organs of WT and CD5L⁻ mice after mid-grade CLP. **a** Representative lung, liver and kidney tissue sections of WT and CD5L⁻ mice 24 and 72 h after mid-grade CLP, stained with hematoxylin and eosin. When present, the histopathological changes observed in the different organs ranged between minimal (lungs and kidney) and mild (liver), not differing significantly from those found in controls. Scale bar: 200 µm. **b** Blind pathology score assessment was performed upon tissue histology analysis. Score 0, 1 and 2 corresponds to absent, minimal and mild alterations, respectively. Animals per group: at 24 h, *n*=7 (WT) and *n*=5 (CD5L⁻), except liver (*n*=7 for WT and *n*=5 CD5L-); at 72 h, *n*=7. **c** Quantification of aspartate aminotransferase (AST, left panel) and creatinine (Cre, right panel) in the blood serum of WT and CD5L⁻ mice 72 h after mid-grade CLP. Statistical comparisons were drawn after performing two-tailed unpaired t-tests with Welch's correction. For AST quantification, *n*=9 (WT) and *n*=8 (CD5L⁻) mice per group. For Cre quantification, *n*=9 (WT) and *n*=7 (CD5L⁻) mice per group **d** *Tnf*, *Il6* and *Il1b* expression was analyzed by RT-qPCR and normalized with *Hprt1* expression in lung, liver and kidney samples from both WT and CD5L⁻ mice, collected 6, 24 and 72 h post after mid-grade CLP. Statistical difference between groups was analyzed by two-way ANOVA with Šídák's multiple comparisons test. *n*= 3 mice/ group. **b-d** Pooled data from at least 2 independent experiments. Floating bars show the minimum, average, and maximum values within each group.

Figure S6

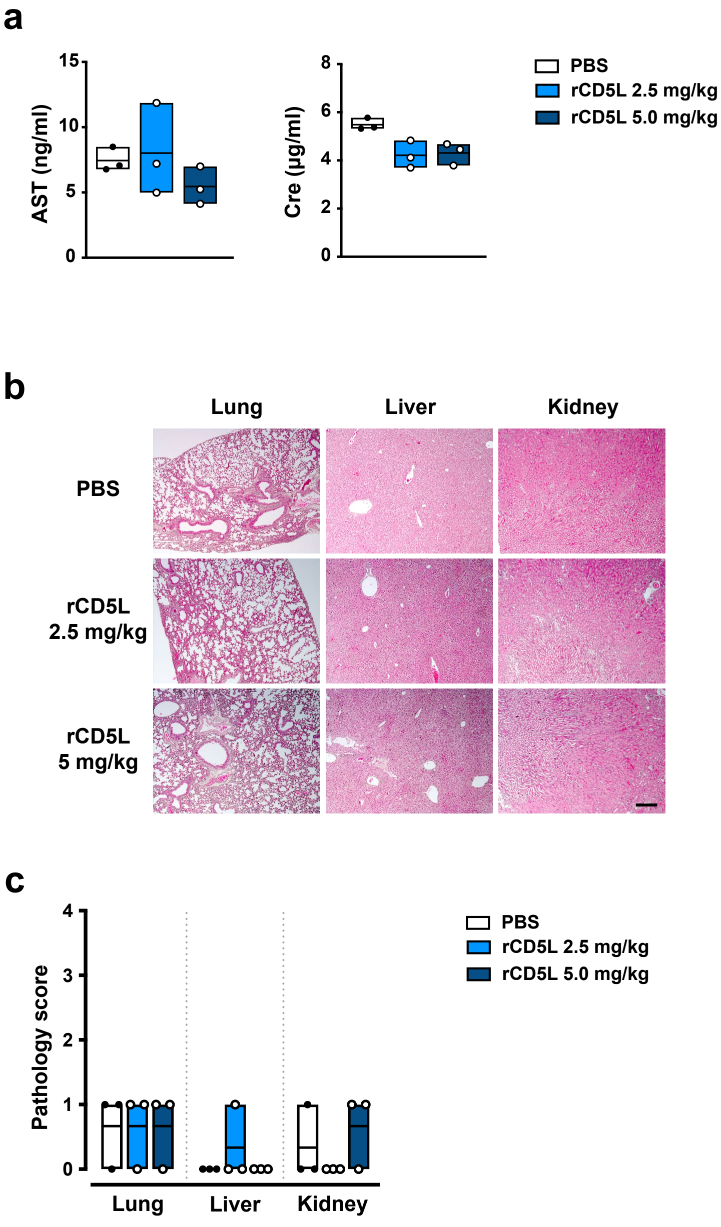

Fig. S6. Absence of histopathological alterations induced by rCD5L IV injection. WT naïve mice were injected IV with 2.5 or 5.0 mg/kg rCD5L, or vehicle (PBS), and relevant organs were collected 48 h later. **a** Quantification of AST (left panel) and creatinine (right panel) in the blood serum of mice. *n*=3 mice per group. **b** Representative lung, liver and kidney tissue sections stained with hematoxylin and eosin. Scale bar: 200 µm. **c** Blind pathology score assessment was performed in the same tissues as in (**b**). Score 0 and 1 corresponds to absent or minimal alterations, respectively. *n*=3 mice per group. **a, c** Statistical comparisons were drawn after performing two-tailed unpaired t-tests with Welch's correction. Floating bars show the minimum, average, and maximum values within each group.

Figure S7

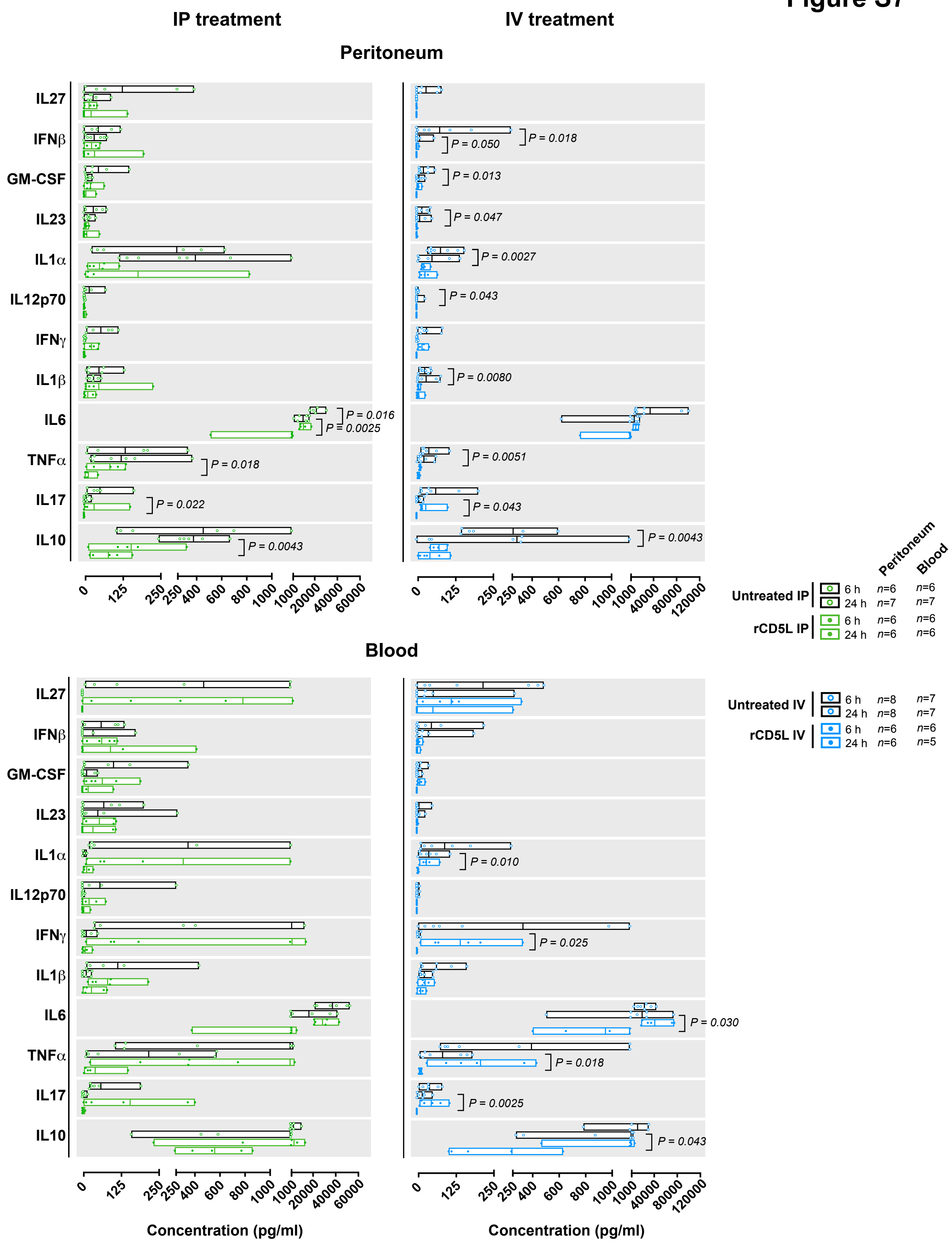
IP treatment
IV treatment
Peritoneum
Blood
IL27
IFNβ
GM-CSF
IL23
IL1α
IL12p70
IFNγ
IL1β
IL6
TNFα
IL17
IL10
P = 0.016
P = 0.0025
P = 0.018
P = 0.022
P = 0.0043
P = 0.050
P = 0.013
P = 0.047
P = 0.0027
P = 0.043
P = 0.0080
P = 0.0051
P = 0.043
P = 0.0043
P = 0.010
P = 0.025
P = 0.030
P = 0.018
P = 0.0025
P = 0.043
0
125
250
400
600
800
1000
20000
40000
60000
80000
120000
Concentration (pg/ml)
Peritoneum
Blood
Untreated IP 6 h n=6 n=6
24 h n=7 n=7
rCD5L IP 6 h n=6 n=6
24 h n=6 n=6
Untreated IV 6 h n=8 n=7
24 h n=8 n=7
rCD5L IV 6 h n=6 n=6
24 h n=6 n=5

Fig. S7. Local and systemic cytokine profiles following rCD5L treatment of WT mice subjected to high-grade CLP. WT mice were subjected to high-grade CLP, and 3 h later injected IP (left panel) or IV (right panel) with 2.5 mg/kg rCD5L, or PBS (untreated). Mice were euthanized 6 h after CLP surgery. For analysis at 24 h, mice were injected IP or IV with 2 doses of 2.5 mg/kg rCD5L, or PBS, at 3 and 6 h after surgery, followed by euthanasia at 24 h. The indicated cytokines were quantified by bead-based multiplex immunoassay on samples from the peritoneal cavity (top panel) and blood serum (bottom panel). Pooled data are from at least 2 independent experiments analyzed by two-tailed Mann-Whitney test. Floating bars show the minimum, average (line), and maximum values. The number of animals (*n*) in each group is indicated.

Figure S8

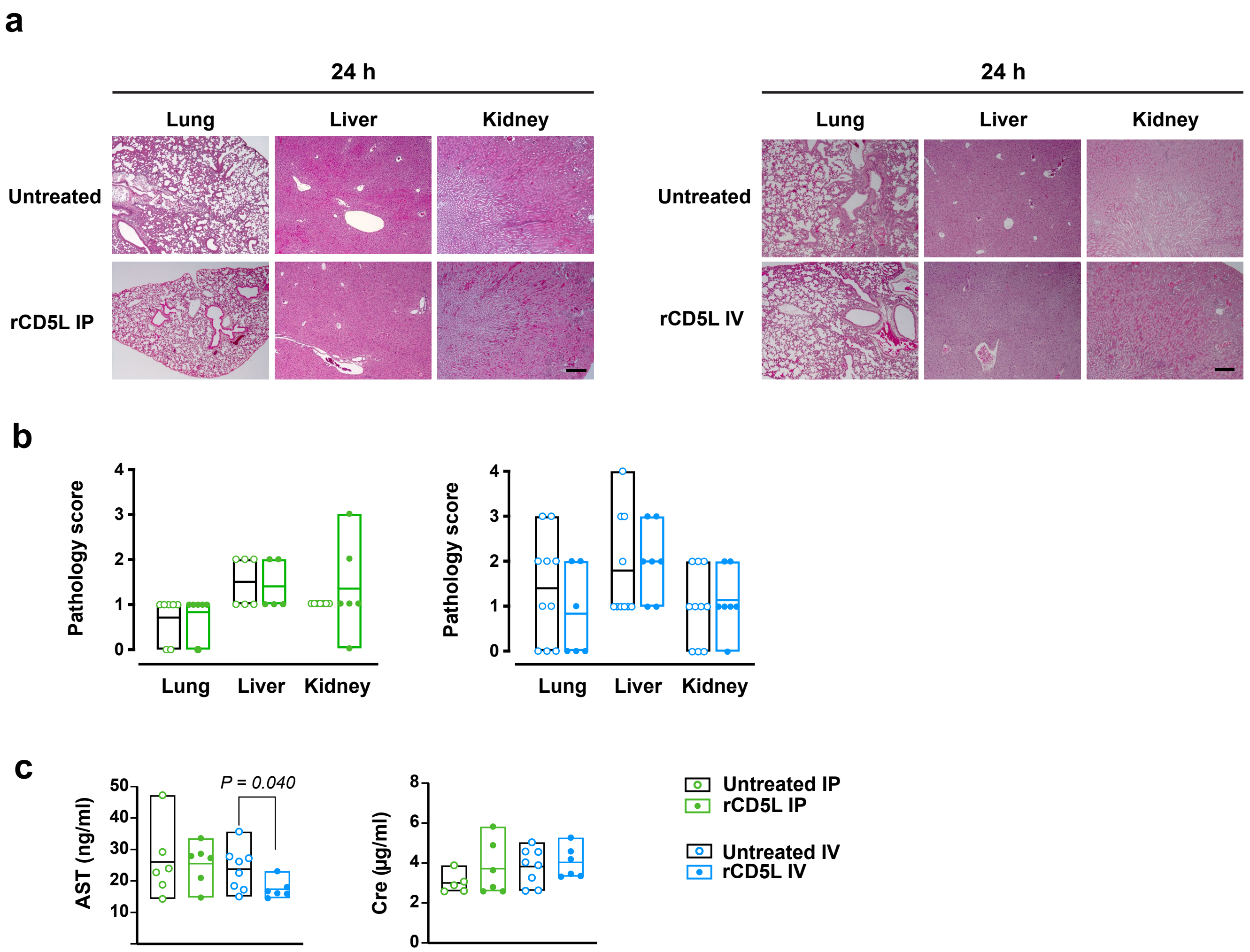

Fig. S8. Pathophysiology analysis of systemic organs after rCD5L treatment of WT mice subjected to high-grade CLP. **a** Representative tissue sections of lung, liver and kidney, 24 h after high-grade CLP, from mice treated with two doses of rCD5L, at 3 and 6 h after surgery, via IP (left panel) or IV (right panel) routes, or injected with PBS (untreated). Sections were stained with hematoxylin and eosin. Scale bar: 200 µm. **b** Blind pathology score assessment was performed upon tissue histology analysis of mice injected IP (left panel) or IV (right panel) with rCD5L. Score 0, 1, 2, 3 and 4 corresponds to absent, minimal, mild, moderate and severe alterations, respectively. Animals per group: IP, *n*=7 (untreated) and *n*=6 (IP treated), except liver (*n*=6 for untreated and *n*=5 for IP treated); IV groups, *n*=10 (untreated) and *n*=7 (IV treated), except lung (*n*=6 for IV treated). **c** Quantification of AST (left panel) and creatinine (right panel) in the blood serum of mice 24 h after CLP. Statistical comparisons were drawn after performing two-tailed unpaired t-tests with Welch's correction. For AST quantification, *n*=6 mice per group except untreated IV (*n*=8). For Cre quantification, *n*=5 (untreated IP), *n*=6 (rCD5L IP), *n*=8 (untreated IV), *n*=6 (rCD5L IV) mice per group. **b-c** Floating bars show the minimum, average, and maximum values within each group. Pooled data from at least 2 independent experiments.

Figure S9

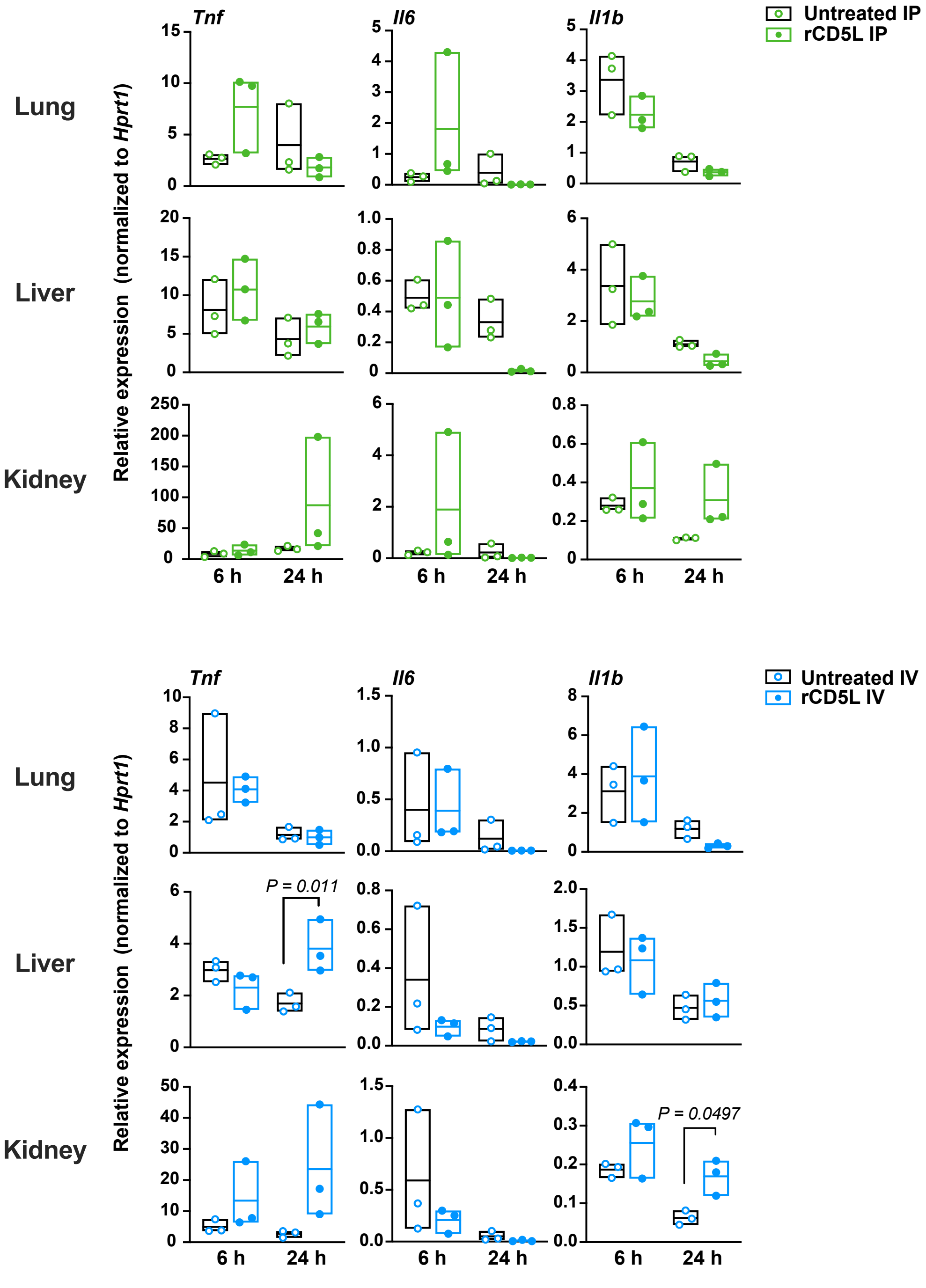

Fig. S9. Inflammatory mediators quantification in systemic organs after rCD5L treatment of WT mice subjected to high-grade CLP. *Tnf*, *Il6* and *Il1b* expression was analyzed by RT-qPCR and normalized with *Hprt1* expression in lung, liver and kidney samples from mice treated IP (upper panel) or IV (lower panel) with rCD5L (2.5 mg/kg/dose), or vehicle (PBS, untreated), injected at 3 h and analyzed at 6 h after CLP or at 3 and 6 h and analyzed at 24 h post surgery. Statistical difference between groups was analyzed by two-way ANOVA with Šídák's multiple comparisons test. *n*=3 mice per group. Floating bars show the minimum, average, and maximum values within each group.

Figure S10

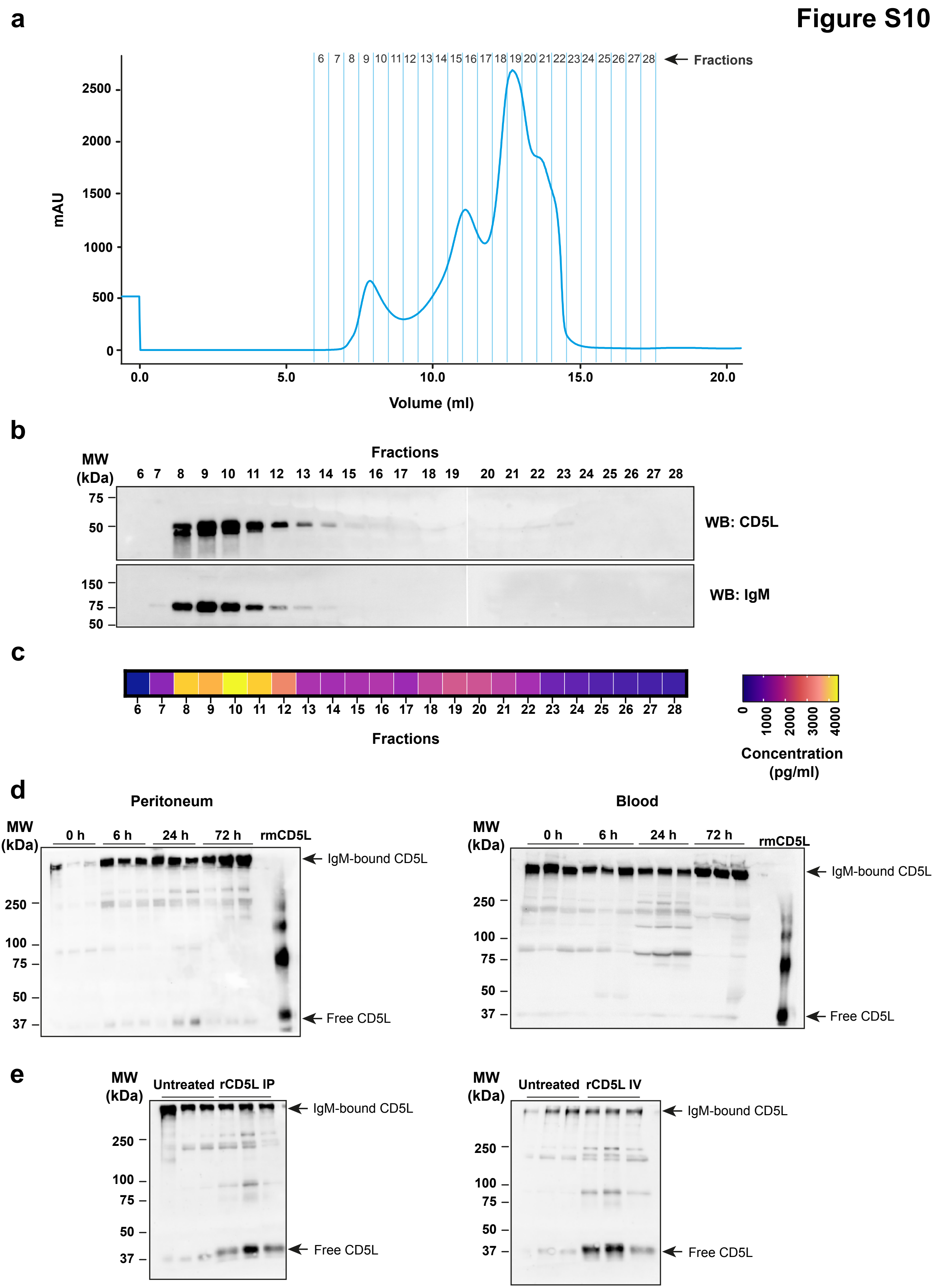
a
Fractions
6 7 8 9 10 11 12 13 14 15 16 17 18 19 20 21 22 23 24 25 26 27 28
mAU
2500
2000
1500
1000
500
0
0.0
5.0
10.0
15.0
20.0
Volume (ml)
b
Fractions
MW (kDa)
6 7 8 9 10 11 12 13 14 15 16 17 18 19 20 21 22 23 24 25 26 27 28
75
50
WB: CD5L
150
75
50
WB: IgM
c
6 7 8 9 10 11 12 13 14 15 16 17 18 19 20 21 22 23 24 25 26 27 28
Fractions
0 1000 2000 3000 4000
Concentration (pg/ml)
d
Peritoneum
MW (kDa)
0 h
6 h
24 h
72 h
rmCD5L
IgM-bound CD5L
250
100
75
50
37
Free CD5L
Blood
MW (kDa)
0 h
6 h
24 h
72 h
rmCD5L
IgM-bound CD5L
250
100
75
50
37
Free CD5L
e
MW (kDa)
Untreated
rCD5L IP
IgM-bound CD5L
250
100
75
50
37
Free CD5L
MW (kDa)
Untreated
rCD5L IV
IgM-bound CD5L
250
100
75
50
37
Free CD5L

Fig. S10. Detection of free and IgM-bound CD5L in the peritoneal fluid and blood of mice subjected to CLP surgery. a Sera from WT mice was loaded onto a Superose 12 column for size exclusion fractionation. The indicated 0.5 ml fractions were chosen based on the elution profile. **b** Fractions 6 to 28 were run on SDS-PAGE, under reducing conditions, and analyzed for the detection of CD5L and IgM by western blotting. **c** CD5L was quantified in the same samples by ELISA. **d** Representative western blots showing the detection of CD5L in peritoneal fluid (left panel) and blood (right panel) samples from 3 mice, collected 6, 24 and 72 h after mid-grade CLP, or from naïve mice (0 h). SDS-PAGE were run under non-reducing conditions to highlight the presence of IgM-bound and free CD5L. **e** Representative western blots showing the detection of CD5L in peritoneal fluid from mice treated IP (left panel) or IV (right panel) with rCD5L (2.5 mg/kg), or injected with PBS (untreated), 3 h after high grade CLP. Samples were collected 3 h later and run under non-reducing conditions. **d, e** *n*=3 mice per group.

Figure S11

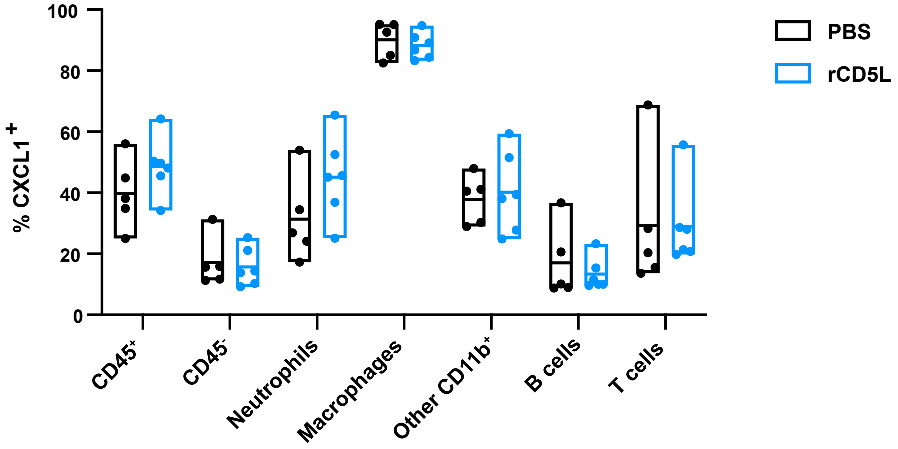

Fig S11. CXCL1 expression by peritoneal cell populations following therapeutic rCD5L administration. CD5L⁻ mice were injected IV with rCD5L (2.5 mg/kg) or vehicle (PBS), 3 h after mid-grade CLP, and peritoneal cells were recovered 3 h later. The percentage of CXCL1-positive cells within leukocytes ($CD45^+$), $CD45^-$ cells, neutrophils ($CD45^+CD11b^+Ly6G^+$), macrophages ($CD45^+CD11b^+F4/80^+$), other $CD11b^+$ ($CD45^+CD11b^+F4/80^-Ly6G^-$), B cells ($CD45^+B220^+$) and T cells ($CD45^+CD3^+$) is represented. Pooled data from 2 independent experiments, analyzed by two-way ANOVA. *n*=5 (PBS) and *n*=6 (rCD5L) mice per group. Floating bars show the minimum, average, and maximum values within each group.
