# Supplementary material for: CD5L as a promising biological therapeutic for treating sepsis
Source: Nat Commun. 2024 May 15;15:4119. doi: 10.1038/s41467-024-48360-8 (PMC11096381; doi:10.1038/s41467-024-48360-8)
Supplement: Supplementary file 3 — Description of Additional Supplementary Files [file 41467_2024_48360_MOESM3_ESM.pdf]

Description of Additional Supplementary Files

File Name: Supplementary Data 1
Description: List of all oligonucleotide sequences used in the study.

File Name: Supplementary Data 2
Description: Populations described by immunophenotyping panels for spleen, peripheral blood, thymus and peritoneal cavity.

File Name: Supplementary Data 3
Description: Panel of antibody clones used in the immunophenotyping panels for spleen, peripheral blood, thymus and peritoneal cavity.

File Name: Supplementary Data 4
Description: List of all antibodies used in the study including clones, catalog number, dilutions and providers.
