# Supplementary material for: CD5L as a promising biological therapeutic for treating sepsis
Source: Nat Commun. 2024 May 15;15:4119. doi: 10.1038/s41467-024-48360-8 (PMC11096381; doi:10.1038/s41467-024-48360-8)
Supplement: Supplementary file 8 — Reporting Summary [file 41467_2024_48360_MOESM8_ESM.pdf]

nature portfolio

Corresponding author(s): Alexandre M. Carmo

Last updated by author(s): Mar 28, 2024

Reporting Summary

Nature Portfolio wishes to improve the reproducibility of the work that we publish. This form provides structure for consistency and transparency in reporting. For further information on Nature Portfolio policies, see our Editorial Policies and the Editorial Policy Checklist.

Statistics

For all statistical analyses, confirm that the following items are present in the figure legend, table legend, main text, or Methods section.

n/a	Confirmed	
☐	☒	The exact sample size (*n*) for each experimental group/condition, given as a discrete number and unit of measurement
☐	☒	A statement on whether measurements were taken from distinct samples or whether the same sample was measured repeatedly
☐	☒	The statistical test(s) used AND whether they are one- or two-sided<br>*Only common tests should be described solely by name; describe more complex techniques in the Methods section.*
☒	☐	A description of all covariates tested
☐	☒	A description of any assumptions or corrections, such as tests of normality and adjustment for multiple comparisons
☐	☒	A full description of the statistical parameters including central tendency (e.g. means) or other basic estimates (e.g. regression coefficient) AND variation (e.g. standard deviation) or associated estimates of uncertainty (e.g. confidence intervals)
☐	☒	For null hypothesis testing, the test statistic (e.g. *F*, *t*, *r*) with confidence intervals, effect sizes, degrees of freedom and *P* value noted<br>*Give P values as exact values whenever suitable.*
☒	☐	For Bayesian analysis, information on the choice of priors and Markov chain Monte Carlo settings
☒	☐	For hierarchical and complex designs, identification of the appropriate level for tests and full reporting of outcomes
☐	☒	Estimates of effect sizes (e.g. Cohen's *d*, Pearson's *r*), indicating how they were calculated

Our web collection on statistics for biologists contains articles on many of the points above.

Software and code

Policy information about availability of computer code

Data collection	FACSDiva 8.0.1, LAS X.
Data analysis	SIMCA Multivariate Data Analysis, FlowJo software (V10.10.0), Tableau Desktop, (ImageJ2 V2.3.0), STAR v2.7.10a, DESeq2 (v.1.34), GraphPad Prism software (v.10.1.1), R (v. 4.1.2) using the Rtsne (v. 0.16) package, ggplot2 (v. 3.4).

For manuscripts utilizing custom algorithms or software that are central to the research but not yet described in published literature, software must be made available to editors and reviewers. We strongly encourage code deposition in a community repository (e.g. GitHub). See the Nature Portfolio guidelines for submitting code & software for further information.

Data

Policy information about availability of data

All manuscripts must include a data availability statement. This statement should provide the following information, where applicable:

- Accession codes, unique identifiers, or web links for publicly available datasets
- A description of any restrictions on data availability
- For clinical datasets or third party data, please ensure that the statement adheres to our policy

RNA seq data has been deposited in European nucleotide archive (ENA) with accession number PRJEB74080. Other data including raw data used to generate plots and the confidence intervals when parametric tests were used to devise statistical significance are also provided in the Source Data file. Source data are provided with this paper.

Human research participants

Policy information about studies involving human research participants and Sex and Gender in Research.

Reporting on sex and gender	N/A
Population characteristics	N/A
Recruitment	N/A
Ethics oversight	N/A

Note that full information on the approval of the study protocol must also be provided in the manuscript.

Field-specific reporting

Please select the one below that is the best fit for your research. If you are not sure, read the appropriate sections before making your selection.

☒ Life sciences ☐ Behavioural & social sciences ☐ Ecological, evolutionary & environmental sciences

For a reference copy of the document with all sections, see nature.com/documents/nr-reporting-summary-flat.pdf

Life sciences study design

All studies must disclose on these points even when the disclosure is negative.

Sample size	No sample-size calculation was performed. The sample sizes were guided by previous studies using similar analyses ((Martinez-Florensa, M. et al. 2016, Català, C. et al. 2022).
Data exclusions	ELISA quantifications and multiplex analysis data were examined using ROUT test and outliers were excluded with the criterion of Q= 1%.
Replication	The number of biological replicates is detailed in figure legends. There were some replicates that were considered irregular and excluded from analysis using ROUT test as described above.
Randomization	Our experimental setup comprises two genotypes (WT and CD5L-) or WT with different treatment schemes. Randomization was performed in assigning animals (WT and CD5L-) to CLP/sham groups and to different time points and assigning WT for the different treatment groups (IP, IV) time points and therapeutic schemes.
Blinding	Investigators were not blinded to group allocation during data collection or analysis. Blinding were not relevant to this study because the groups are defined by genotype and treatment application. Histopathology evaluation was conducted by a pathologist blinded to the experimental conditions

Reporting for specific materials, systems and methods

We require information from authors about some types of materials, experimental systems and methods used in many studies. Here, indicate whether each material, system or method listed is relevant to your study. If you are not sure if a list item applies to your research, read the appropriate section before selecting a response.

Materials & experimental systems

n/a	Involved in the study	
☐	☒	Antibodies
☒	☐	Eukaryotic cell lines
☒	☐	Palaeontology and archaeology
☐	☒	Animals and other organisms
☒	☐	Clinical data
☒	☐	Dual use research of concern

Methods

n/a	Involved in the study	
☒	☐	ChIP-seq
☐	☒	Flow cytometry
☒	☐	MRI-based neuroimaging

Antibodies

Antibodies used	Specificity | Clone | Catalog number | Dilution | Provider

- Immunophenotyping-Thymus
CD3e 2C11 745836 100 eBioscience
CD4 RM4.5 563151 200 BD Biosciences
CD5 53-7.3 562739 400 BD Biosciences
CD8a 53-6.7 564297 100 BD Biosciences
CD24 M1.69 564237 400 BD Biosciences
CD25 PC61 BLE102026 100 Biolegend
CD27 LG.3A10 558754 100 BD Biosciences
CD44 IM7 BLE103043 200 Biolegend
CD69 H1.2F3 563290 50 BD Biosciences
CD71 R17217 BLE113812 100 Biolegend
CD117 2B8 562417 400 BD Biosciences
CD161 PK136 550627 200 BD Biosciences
TCRd GL3 15-5711-82 200 eBioscience
QA-2 695H1.9.9 BLE121710 200 Biolegend

- Immunophenotyping-Spleen

CD3e 2C11 745836 200 BD Biosciences
CD4 RM4.5 563151 200 BD Biosciences
CD5 53-7.3 562739 200 BD Biosciences
CD8 53-6.7 564297 100 BD Biosciences
CD11b M1.70 563168 200 BD Biosciences
CD11c HL3 564080 50 BD Biosciences
CD19 1D3 562956 200 BD Biosciences
CD44 IM7 553133 400 BD Biosciences
CD62L MEL14 BLE104428 800 Biolegend
CD161 PK136 564143 100 BD Biosciences
CD317 927 25-3172-82 800 eBioscience
Ly6C AL21 562728 200 BD Biosciences
Ly6G 1A8 562737 800 BD Biosciences
TCRd GL3 15-5711-82 400 eBioscience
F4/80 BM8 BLE123110 200 Biolegend
MHCII M5-114.15.2 BLE107622 1600 Biolegend
IgD 11-26c.2A 560668 200 Biolegend

- Immunophenotyping-Peritoneum

CD011c HL3 564986 50 BD Biosciences
LY6g 1A8 562737 200 BD Biosciences
CD005 53-7.3 562739 200 BD Biosciences
MHCII M5/114.15.2 BLE107635 200 Biolegend
CD117 2B8 563146 100 BD Biosciences
CD161 PK136 564143 50 BD Biosciences
CD011b M1/70 563168 100 BD Biosciences
LY6c AL-21 553104 100 BD Biosciences
CD115 AFS98 46-1152-82 50 ThermoFisher Sc
CD192 SA203G11 BLE150610 50 Biolegend
SiglecF E50-2440 562757 200 BD Biosciences
CD19 1D3 15-0193-82 400 ThermoFisher Sc
F4/80 BM8 BLE123114 800 Biolegend
CD064 X54-5/7.1 BLE139306 100 Biolegend

-Immunophenotyping - PBL

CD5 53-7.3 561244 400 BD Biosciences
CD8a 53-6.7 563068 100 BD Biosciences
CD19 6D5 BLE115539 400 Biolegend
CD11b M1/70 553310 800 BD Biosciences
CD44 IM7 553134 1000 BD Biosciences
CD45 30F11 35-0451-82 400 ThermoFisher Sc
CD4 RM4-5 552775 200 BD Biosciences
CD161 PK136 550627 100 BD Biosciences
Ly6G 1A8 561236 200 BD Biosciences
Ly6C HK1.4 BLE128026 3200 Biolegend

Other analysis

CD45-PerCP/Cy5.5 I3/2.3 147705 400 Biolegend
CD11b-PE M1/70 101208 200 Biolegend
CD11b FITC M1/70 101205 200 Biolegend
Ly6G-PB 1A8 127612 100 Biolegend
Ly6G-APC 1A8 127613 400 Biolegend
CD11c-PB N418 117322 100 Biolegend
F4/80- APC-Cy7 BM8 123117 100 Biolegend
F4/80- PE BM8 123109 200 Biolegend

Ly6C-FITC HK1.4 128005 100 Biolegend
CD206- PE C068C2 141705 200 Biolegend
CD19-APC 6D5 115511 400 Biolegend
CD5-PE/Cy7 53-7.3 100621 200 Biolegend
CD3-APC/Cy7 17A2 100222 100 Biolegend
B220-PE RA3-6B2 103208 200 Biolegend
CD16/32 antibody 93 101320 50 Biolegend
F480 BM8 123101 100 Biolegend
HMGB1 3E8 651401 100 Biolegend
Tetra His 34670 5000 Qiagen
CXCL1 1174A MAB4532-SP 100 R&D
CD5L Goat polyclonal AF2834 1500 (for western blot); 200 (flow cytometry) R&D

-Secondary Antibodies

Specificity | Catalog number | Dilution | Provider

AlexaFluor 488-conjugated donkey anti-goat IgG A11055 500 Life Technologies
AlexaFluor 568-conjugated goat anti-mouse IgG A11004 500 Life Technologies
AlexaFluor 594-conjugated goat anti-rat IgG 405422 500 Biolegend
AlexaFluor 647-conjugated goat anti-rabbit IgG A21244 500 Life Technologies
Unconjugated goat anti-mouse Ig 1010-01 3 µg/mL SouthernBiotech
HRP-labeled goat anti-mouse IgM 1021-05 5000 SouthernBiotech
HRP-labeled donkey anti-goat IgG sc-2020 10000 Santa Cruz Biotechnology
HRP-labeled goat anti-mouse IgG 405306 10000 Biolegend

Validation

All antibodies used here are commercially available. Antibodies for flow cytometry purchased from BioLegend, BD Biosciences, eBioscience and ThermoFisher Sc are quality control tested by immunofluorescent staining with flow cytometric analysis as mentioned on the manufacturer's website. Antibodies for immunofluorescence purchased from Biolegend (F4/80 and HMGB1) are quality controled by western blotting and flow cytometry and verified for the application in which they were used by reference on the manufacturer's website. Tetra his (Qiagen) are quality controled by western blotting as mentioned on the manufacturer's website. Anti-CXCL1 antibody (R&D) is verified for Intracellular Staining by Flow Cytometry as reported on the manufacturer's website. Anti-CD5L (R&D) is verified for ELISA, western blotting and immunohistochemistry as indicated on the manufacturer's website.

Animals and other research organisms

Policy information about studies involving animals; ARRIVE guidelines recommended for reporting animal research, and Sex and Gender in Research

Laboratory animals

All experiments were conducted in 8- to 12-week-old C57BL/6J mice.
Mice were kept in an AAALAC-accredited animal facility with high standard accommodation. All cages contained corncob bedding (LBS serving Biotechnology, United Kingdom), absorbent paper (Renova, Portugal) for nesting material, and a cardboard tube (LBS serving Biotechnology, United Kingdom). Mice had access to Teklad Harlan 2014S (Envigo, United Kingdom) chow and tap water ad libitum. Room temperature was maintained at 20-24°C with a relative humidity of 45-65 %. Mice were housed under a 12:12 hour dark/light cycle with lights on between 08:00 h and 20:00 h.

Wild animals

No wild animals were used in the study.

Reporting on sex

Female mice were used in therapeutic experiments, due to their significant lower weight compared to males, thus requiring a smaller amount of rCD5L to be administered. There have been mixed reports on how sex affects sepsis in clinical and rodent sepsis models, with most reporting improved survival in females (Cerceo E. et al, 2021; Wehrenpfennig, P. et al, 2014). We did not find a gender bias effect in our experimental conditions, in line with a recent report that included a large cohort of mice (Garcia, L.F. et al, 2023), therefore both males and females were used in our studies.

Field-collected samples

No field collected samples were used in the study.

Ethics oversight

All experiments were conducted in 8- to 12-week-old male and female C57BL/6J mice purchased from Charles River and bred in-house following Portuguese (Portaria 1005/92) and European (Directive 2010/63/EU) legislations concerning housing, husbandry and welfare. The project was reviewed and approved by the Ethics Committee of the Instituto de Investigação e Inovação em Saúde (i3S), Universidade do Porto, and by the Portuguese National Entity Direção Geral de Alimentação e Veterinária (license reference: 009951).

Note that full information on the approval of the study protocol must also be provided in the manuscript.

Flow Cytometry

Plots

Confirm that:

☒ The axis labels state the marker and fluorochrome used (e.g. CD4-FITC).

☒ The axis scales are clearly visible. Include numbers along axes only for bottom left plot of group (a 'group' is an analysis of identical markers).

☐ All plots are contour plots with outliers or pseudocolor plots.

☒ A numerical value for number of cells or percentage (with statistics) is provided.

Methodology

Sample preparation	Cells were recovered and 1 x 10^6 cells were washed with PBS before staining with a viability dye (Fixable viability dye, eBioscience). After, cells were kept in flow staining media (FSM, PBS containing 2% FBS) and Fc receptors were blocked (TruStain FcX anti-mouse CD16/32 antibody) before staining with fluorochrome-conjugated antibodies against surface markers or anti-CD5L unconjugated antibody followed by secondary antibody (Table S4).
Instrument	BD LSRFortessa; FACSCanto II; BD Accuri C6
Software	BD FACSDiva 8.0.1; FlowJo V10.10.0.
Cell population abundance	*Describe the abundance of the relevant cell populations within post-sort fractions, providing details on the purity of the samples and how it was determined.*
Gating strategy	*Describe the gating strategy used for all relevant experiments, specifying the preliminary FSC/SSC gates of the starting cell population, indicating where boundaries between "positive" and "negative" staining cell populations are defined.*

☐ Tick this box to confirm that a figure exemplifying the gating strategy is provided in the Supplementary Information.
